# Trends of chronic illness in emergency department admissions among elderly adults in a tertiary hospital over ten years

**DOI:** 10.1186/s12913-021-07309-z

**Published:** 2021-12-04

**Authors:** Zhongxun Hu, Fahad Javaid Siddiqui, Qiao Fan, Sherman W. Q. Lian, Nan Liu, Marcus E. H. Ong

**Affiliations:** 1 Duke-NUS Medical School, Singapore, Singapore; 2 Prehospital and Emergency Research Centre, Health Services & Systems Research, Duke-NUS Medical School, Singapore, Singapore; 3 Center for Quantitative Medicine, Duke-NUS Medical School, Singapore, Singapore; 4 Department of Emergency Medicine, Singapore General Hospital, Singapore, Singapore; 5 Health Services & Systems Research, Duke-NUS Medical School, Singapore, Singapore; 6 Department of Emergency Medicine, Singapore General Hospital, Singapore, Singapore; 7 Health Services and Systems Research, Prehospital and Emergency Research Center, Duke-NUS Medical School, Singapore, Singapore

**Keywords:** Aging population, Chronic illness, Emergency admission, Health services

## Abstract

**Background:**

This study aimed to determine to what extent an aging population and shift to chronic illness has contributed to emergency admissions at a tertiary care hospital over ten years.

**Methods:**

This was a retrospective observational study performed using a database of all emergency admissions from the Emergency Department (ED) at a single tertiary hospital in Singapore during a ten-year period (January 1st, 2008 to December 31st, 2017). Emergency admissions were defined as ED visits with inpatient admission as the disposition. This study analyzed the trends of demographics, pre-existing comorbidities, chronic conditions or ambulatory care sensitive conditions (ACSC) of all patients who underwent emergency admissions in Singapore General Hospital.

**Results:**

A total of 446,484 emergency records were included. For elderly patients, the proportions of them had pre-existing multimorbidity at the time of undergoing emergency admissions were found to be lower at the end the 10-year study period relative to the beginning of the study period. The proportions of emergency admissions whose ED primary diagnoses were categorized as chronic conditions and certain chronic ACSC including chronic obstructive pulmonary disease, congestive heart failure, diabetes complications, and epilepsy also decreased for elderly patients over the 10-year study period.

**Conclusions:**

In Singapore, despite a rapidly aging population, there have been surprisingly lower proportions of chronic conditions, pre-existing comorbidities, and chronic ACSC among the elderly emergency admissions. This is possibly consistent with an overall improved management of the chronic conditions among the elderly population. Future studies should include similar studies at the national level and comparison with other healthcare settings in different countries.

**Supplementary Information:**

The online version contains supplementary material available at 10.1186/s12913-021-07309-z.

## Background

Worldwide, the pace of population aging is increasing, both in developed and developing countries [1]. As people live longer, multimorbidity also becomes increasingly common, which is associated with increased use of inpatient and outpatient care [2, 3]. Singapore is facing one of the fastest aging populations in the world. The proportion of the elderly population is expected to rise from 9% in 2010 to 22.5% in 2030 [4].

.Like many other countries, Singapore is challenged by rising demand for emergency care, evidenced by a 47% increase in annual total visits to public sector emergency departments (EDs) from 2005 to 2015 [5]. A significant portion of ED visits goes on to eventually become emergency admissions. Emergency admissions, defined as patients visiting ED admitted for inpatient care, constitute a significant policy concern. While inpatient care can be effective, it can be costly and may not be in the best interest of the patients when ambulatory care is possible. In particular, when elderly patients (age 65 and above) visit the ED, they are more likely to have emergency admissions compared to younger patients [6, 7]. Additionally, elderly patients are more likely to have a longer hospital stay, higher healthcare costs, and higher overall mortality rate after emergency admissions [8–10].

.Although many drivers for increased ED visits have been well described [10–13], those for emergency admissions specifically have not. There has been evidence showing that a higher Charlson Comorbidity Index is associated with an increased rate of emergency admissions [5, 11]. Wittenberg et al. have argued that proximity to the end of life, instead of older age per se, is associated with a higher rate of emergency admission [14]. A subgroup of potentially preventable emergency admissions can be studied by ambulatory care sensitive conditions. Ambulatory care sensitive conditions (ACSC) [15] have been commonly used as an indicator of potentially preventable hospital admissions and a proxy measure of the quality and accessibility of primary care. There have been several trend analyses of ACSCs in different parts of the world. However, the results are far from universal, and the trends vary significantly by country and by specific condition [16–21].

.The overall objective of this study is to determine to what extent the aging population and chronic conditions have contributed to the volume of elderly emergency admissions. It was hypothesised that an aging population with an increasing number of chronic conditions [22] was associated with a higher number of emergency admissions. We aim to test this hypothesis by analysing the trends of proportions of chronic conditions, number of pre-existing comorbidities, and chronic ACSCs independently, using a ten-year, comprehensive single-centre database.

## Methods

### Study design

This was a retrospective observational, single-centre study. This study was approved by Singapore Health Services’ Centralized Institutional Review Board.

### Study setting and population

Singapore has a mixed healthcare delivery system consisting of both public and private providers, with the public sector handling over 80% of the population’s healthcare needs [23]. There were a total of 5 adult care public hospital EDs in Singapore in 2008. Subsequently, two additional EDs started operations in 2010 and 2015 respectively with the opening of two new public hospitals, bringing the total number of EDs to 7 in 2017.

The public healthcare system in Singapore consists of three integrated clusters. The study hospital, Singapore General Hospital (SGH), is part of the Singapore Health Services (SingHealth) cluster, which delivers healthcare predominantly for the population in the Eastern region. We performed the study using a database from the SGH, the largest and oldest tertiary medical centre in Singapore, with comprehensive clinical services and over 1700 inpatient beds. Annually, the SGH ED receives more than 120,000 ED visits, over 40,000 of which are converted to inpatient admissions. As a percentage of the total emergency department visits of all public hospitals in Singapore, SGH ED visits represented 17% in 2008 and 13% in 2017. The analysis was based on extracted data from SGH’s electronic medical health system, namely Singhealth Electronic Health Intelligence System (eHints). Detailed information from other public or private hospitals were not available. The data was recorded as per individual emergency admission episodes. Multiple emergency admission episodes from the same patient are considered separate individual episodes. All patients who underwent emergency admissions at SGH from 1 January 2008 to 31 December 2017 were included in this study. Patients at SGH below the age of 18 were excluded.

### Measures

Selected variables included two demographic variables, three administrative variables, and 18 clinical variables. Demographic variables included age and postal code. ED administrative variables included anonymized case identification number, anonymized admission number, and ED registration date. Clinical variables included the presence of 17 comorbidities from the past 5 years of hospital discharge records before the index emergency admission, and primary ED diagnosis. Patients’ identifying information was removed to ensure anonymity.

### Identification of pre-existing comorbidities

The definitions of comorbidities employed in this study were based on the Charlson Comorbidity Index [24]. The 17 comorbidities defined in this study included prior myocardial infarction, congestive heart failure, peripheral vascular disease, cerebrovascular disease, dementia, chronic pulmonary disease, rheumatological disease, peptic ulcer disease, mild liver disease, diabetes, cerebrovascular (hemiplegia) event, moderate to severe renal disease, diabetes with chronic complications, cancer without metastases, moderate to severe liver disease, metastatic solid tumour, and acquired immune-deficiency syndrome (AIDS). Pre-existing comorbidities were determined from their past 5 years of hospital discharge records before the referenced emergency admission. The number of pre-existing comorbidities was further grouped into three categories – no pre-existing comorbidity, single pre-existing comorbidity, and pre-existing multimorbidity in which the patient had two or more of the comorbidities. For our dataset, the information needed to trace 5 years back in their medical records was only available from 2012 onwards. Therefore, the timeline for this analysis only included 2012-2017.

### SNOMED CT to ICD-10 conversion for primary ED diagnosis

Our eHints dataset recorded primary ED diagnoses according to the International Classification of Diseases Version 9 (ICD-9) from 2008 to 2014. From 2015 to 2017, the EHR switched to Systematized Nomenclature of Medicine Clinical Terms (SNOMED CT) for the computerized coding of ED diagnoses. To facilitate consistent comparison of primary ED diagnoses across the study period, SNOMED CT codes were first converted to ICD-10 using SNOMED CT to ICD-10-CM Map released by the National Library of Medicine and ICD-10-CM Official Guidelines for Coding and Reporting [25, 26].

### Identification of chronic conditions in primary ED diagnosis

To determine whether the primary ED diagnosis would be categorized as a chronic condition or non-chronic condition, we adapted Chronic Condition Indicator (CCI) for ICD-9 [27] and ICD-10 [28], respectively, developed by Agency for Healthcare Research and Quality. A chronic condition is defined as a condition expected to last 12 months or longer and results in functional limitations and or the need for ongoing medical intervention [29]. Therefore, each emergency admission episode’s primary ED diagnosis can be designated either chronic or not chronic based on its ICD-9 or ICD-10 code.

### Identification of ambulatory care sensitive conditions (ACSC) in primary ED diagnosis

Emergency admissions for ACSC were identified from the ICD-9 or ICD-10 primary ED diagnoses. The lists of diagnosis codes adopted in this study were based on the lists described by Billings et al. [15] and the subsequent ICD-10 version [30]. The algorithm detects 24 ACSC conditions. ACSC conditions are further categorised into acute, chronic, and avoidable conditions. This study focused on the nine chronic ACSC (Table S1).

### Statistical methods

Data wrangling and analysis were performed using R version 4.0.2 (R Foundation, Vienna, Austria). Chronic conditions, number of pre-existing comorbidities, and chronic ACSCs were analysed as proportions of the total SGH ED admissions. Proportion estimates in a given year were reported with 95% confidence interval. Mann-Kendall test (MK) was used to statistically assess whether there is a monotonic upward or downward trend of a proportion over time. A monotonic upward or downward trend means the proportion consistently increased or decreased over time, but the trend may not necessarily be linear. Additionally, a modified Mann-Kendall test (MMKH) using Hamed and Rao variance correction approach [31] was performed on trend analysis to address the issue of serial correlation. The serial correlation was evaluated using the *acf* function in R. The serial correlation (at lag 0-20) was detected in monthly aggregated data. Thus we performed the MMKH test for the monthly aggregated analysis. For annually aggregated data with much fewer time points, the serial correlation was negligible. Trend analysis was performed on the proportions of elderly emergency admissions with pre-existing multimorbidity at the time of admission, whose primary ED diagnosis was categorized as chronic conditions, and whose primary ED diagnosis was identified as ACSC.

## Results

### General trends

There were 446,484 emergency admission episodes included in the analysis from 2008 to 2017. The emergency admission patient population at SGH represents a wide geographical distribution across the country (Fig. S1), with a higher concentration in the immediate neighbourhood Bukit Merah and the eastern region in Singapore, which correlates well with the coverage area of the Singhealth healthcare cluster [32].

**Fig. 1:**
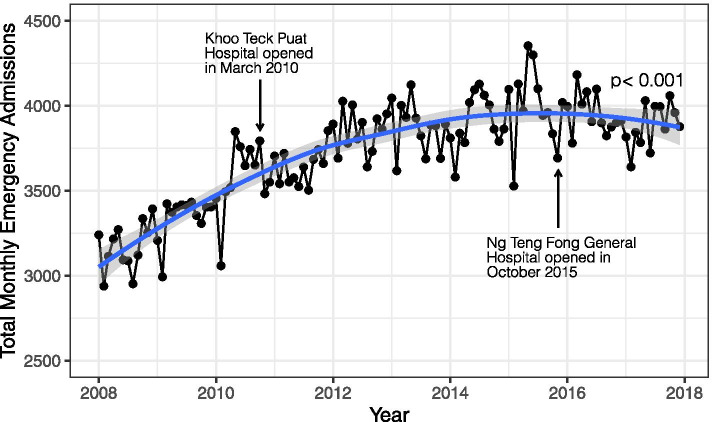
Monthly number of emergency admissions at SGH from 2008 to 2017 (Regression line with 95% CI was plotted using LOESS (locally estimated scatterplot smoothing). Mann-Kendall test was performed to assess the presence of monotonic trend of monthly number (*p* < 0.001))

.The average monthly number of emergency admissions at SGH increased by 22%, from 3204 in 2008 to 3902 in 2017. MMKH was performed on the trend of monthly numbers (*p* < 0.001). From 2008 to 2017, the year-on-year increase averaged at 3.4%. Despite the overall upward trend, the average monthly number started to plateau from 2015 (Fig. 1).

From 2008 to 2017, the proportion of elderly in the Singapore population increased from 8.7 to 13.1% [33]. The aging population is reflected in the trend of the proportion of elderly in the SGH emergency population, which increased from 46 to 53% during the same period.

### Trends of pre-existing comorbidity

**Fig. 2:**
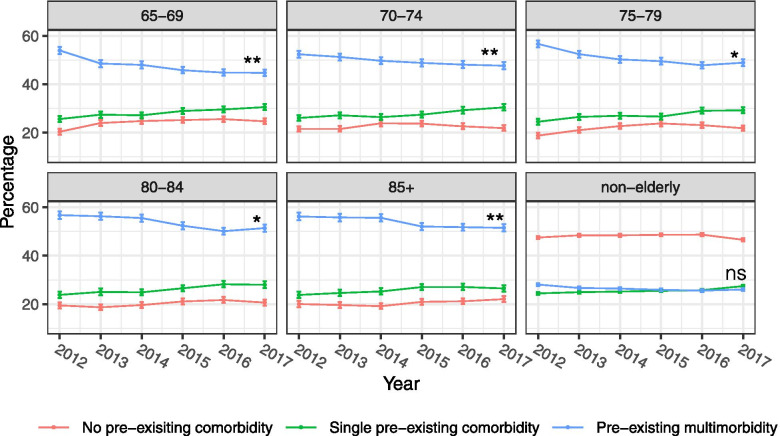
Trends of number of pre-existing comorbidities at the time emergency admissions by age group from 2012 to 2017 (Mann-Kendall test was performed to assess the presence of a monotonic trend in the proportions of pre-existing multimorbidity. (ns: not statistically significant, *: *p* < 0.05, **: *p* < 0.01))

The proportion of emergency admission patients at SGH with multimorbidity stayed constant for the non-elderly population but decreased for all age groups in the elderly population from 2012 to 2017 (Fig. 2). This number has also been consistently higher in the elderly population than in the non-elderly population.

### Trends of chronic conditions

**Fig. 3:**
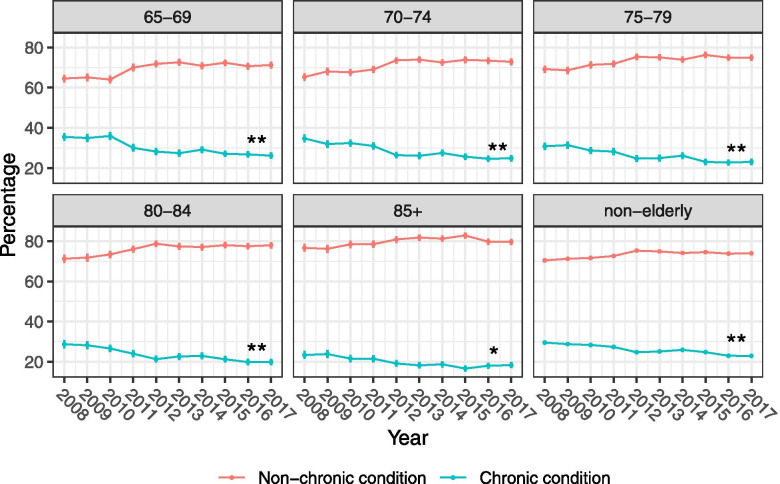
Trends of chronic conditions at the time emergency admissions by age group from 2008 to 2017 (Mann-Kendall test was performed to assess the presence of a monotonic trend in the proportions of chronic conditions. (ns: not statistically significant, *: *p* < 0.05, **: *p* < 0.01))

There were statistically significant downward trends for the proportions of chronic conditions as the ED primary diagnosis for SGH emergency admissions from 2008 to 2017 across all age groups (Fig. 3).

### Trends of chronic ambulatory care sensitive conditions (ACSC)

**Fig. 4:**
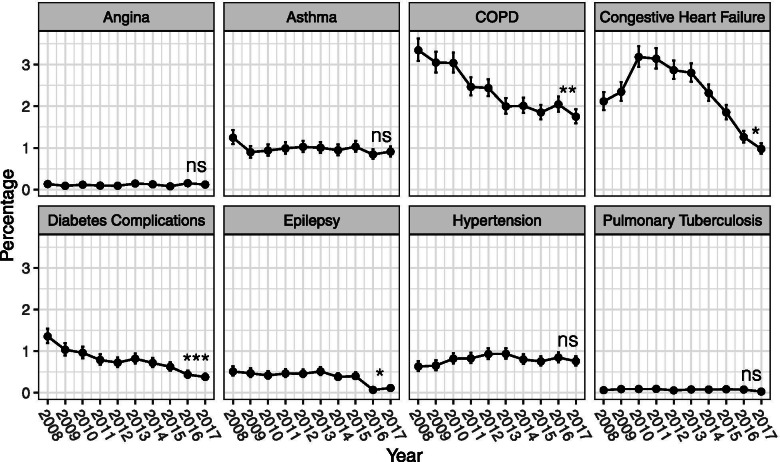
Trends of chronic ACSC as primary ED diagnosis for emergency admissions by condition from 2008 to 2017 (Mann-Kendall test was performed to assess the presence of a monotonic trend in the proportions of chronic ACSCs. (ns: not statistically significant, *: *p* < 0.05, **: *p* < 0.01, ***: *p* < 0.001))

Of the eight chronic ACSCs included, 4 of them – COPD, congestive heart failure, diabetes complications, and epilepsy - had statistically significant reductions in their proportions as the ED primary diagnosis of SGH elderly emergency admissions from 2008 to 2017. The rest did not show any statistically significant monotonic trends, although these conditions had very low proportions (less than 1.5%), to begin with, in 2008 (Fig. 4).

## Discussion

In this study, we demonstrated that chronic conditions as a whole showed a relative decline in elderly patients who underwent emergency admissions. In comparison with a United Kingdom (UK) study, the findings of a substantial reduction in congestive heart failures are consistent with our findings. However, the UK study reported a significant increase in emergency admissions for COPD, diabetes complications and epilepsy, for which we have observed the opposite trend [17]. The marked variation in the trends among countries can result from different epidemiology and healthcare approaches in managing these conditions. Taken together, we found that chronic conditions have not been a major driver in the increasing number of emergency admissions in Singapore. However, the heterogeneity of the impact from an aging population is a reminder that context-specific analysis is needed.

Several factors could have contributed to reducing the proportions of emergency admissions due to chronic conditions among the elderly population. Singapore has made efforts in the last decade to improve the quality and accessibility of ambulatory care, including primary care and outpatient specialist care. A notable example is CHAS (Community Health Assist Scheme), which subsidizes Singapore Citizens for chronic medical care at private General Practioner (GP) clinics as well as public Specialist Outpatient Clinics (SOCs) [34]. Since its inception in 2009, CHAS has been iteratively enhanced to be more inclusive in terms of patient demographics and eligible conditions. Another example targeted at the elderly population is the Pioneer Generation Package introduced in 2014, which provides substantial subsidies for outpatient bills and disabilities assistance to Singapore citizens born before 1949 [35]. Moreover, community care services such as Home Care, Day Care, Stay-In Care have been expanded support homebound or chronically ill seniors in the community [36]. Additional policies such as Family Medicine Clinics and Primary Clinic Networks models have also been introduced to improve chronic disease management [37].

.From the ED’s perspective, a possible explanation for the observed trends is that the ED has been more effective in gatekeeping unnecessary emergency admissions. Locally, there has been a move towards ambulatory management for specific conditions or procedures rather than admission. To test this hypothesis, an overview of death rates due to chronic conditions among the elderly could be helpful. Such data are available from the Department of Statistics for heart and hypertensive diseases as well as malignant neoplasms as the causes of death [38]. The annual death rates due to heart and hypertensive diseases for the elderly in Singapore, defined as deaths per 1000 residents aged 65 and above, have steadily decreased from 8.8 in 2008 to 6.6 in 2017. Similarly, the annual death rates due to malignant neoplasms have decreased from 9.4 in 2008 to 7.5 in 2017. The trends for these death rates are not necessarily inconsistent with the “gatekeeping” hypothesis. Although, other alternative explanations include a decreasing prevalence of these conditions and the reduced death rates as a result of better chronic management of these conditions. These alternative hypotheses would require additional studies to be confirmed.

A few potential confounding factors might have contributed to the observed trends but are difficult to account for due to the limited availability of the data. First, trends in community-wide prevalence and fatality rate of the same chronic conditions studied could provide more context for our data. Such context can suggest whether the observed trends result from the shifting epidemiology in the wider community or more as a result of the changes in healthcare delivery. Second, Singapore has been increasing the capacity of public hospitals by the progressive opening of new hospitals. Changes in the admissions profiles in other public hospitals due to redistribution of patients could also result in changes in admission trends in SGH. Third, over the past two decades, there have been numerous incremental advances in the medical management of chronic conditions. However, the effects of these advances are difficult to assess quantitatively. Fourth, there could be emergency admissions for non-medical reasons. For example, some patients could have been admitted from the ED to claim insurance for their medical bills, even when the medical indications for admission were not met.

Elderly patients pose special diagnostic and management challenges to ED physicians due to multimorbidity, polypharmacy, atypical presentations for dangerous conditions, and various social issues [39]. Practice thresholds for admissions are directly related to admission rates, which has been described in the United States [40] and the United Kingdom [41]. Looking forward, the ED, along with the rest of the healthcare system, will need to transform together to manage a rapidly aging population. This exploratory study has highlighted the encouraging trends for emergency admissions among the elderly population in the primary study hospital. To identify concrete policy implications, further studies need to establish more evident causal relationships between the trends and the possible causes.

## Limitations

Our study has several potential limitations. First, the study was done with a single-centre dataset, which limits the generalizability of its conclusions. As a tertiary academic medical centre, SGH might receive different epidemiology compared to the general population. Secondly, as a retrospective observational study using administrative data, the accuracy of the results is susceptible to the bias created by information recording. For example, over the 10-year study period, ED physicians may have changed their preferences to record certain diagnoses as the primary diagnosis over secondary diagnosis, or vice versa. The switch from ICD-9 to SNOMED CT in 2015 as the administrative coding system could also have introduced bias despite the best efforts in grouping diagnoses into broad categories. However, it is worth noting that few of the trends described started to change in 2015, mitigating this concern. Thirdly, this study only included admitted patients. However, we are planning a follow-up study describing the epidemiology of all ED visits during the period, including discharged patients, which will provide us with a further understanding of the trends noted in our analysis. Finally, we did not look at how ED frequent flyers contributed to the trends in this analysis. Our primary outcome was the trend of ED admissions, regardless of the contribution of frequent visitors on these admissions. A separate analysis of frequent users of the ED is planned for a follow-up study.

## Conclusions

We found that despite an higher proportions of the elderly in emergency care, there have been surprisingly fewer chronic conditions, pre-existing comorbidities, and chronic ACSC among the elderly emergency admissions in SGH ED. In Singapore, policies to improve access and quality of ambulatory care and other public health efforts have possibly helped to reduce the demand for emergency admissions. It will be interesting to perform similar studies at the national level and compare with other healthcare settings in different countries in future studies.

## Supplementary Information

**Additional file 1: Table S1.** List of chronic ACSC and the ICD-9 and ICD-10 Codes. Description of data: List of ICD-9 ad ICD-10 codes used to identify chronic ACSC. **Figure S1.** Geographical distribution of SGH emergency admission patients according to planning areas in Singapore. Description of data: Numbers show the total number of patients from the specific planning area from 2008 to 2017.

## Data Availability

The data that support the findings of this study are available from Singapore Health Services but restrictions apply to the availability of these data, which were used under license for the current study, and so are not publicly available. Data are however available from the authors upon reasonable request and with permission of Singapore Health Services.

## Abbreviations

ACSC: Ambulatory care sensitive conditions
AIDS: Acquired immunodeficiency syndrome
CHAS: Community Health Assist Scheme
COPD: Chronic obstructive pulmonary disease
ED: Emergency Department
GP: General practitioner
ICD-9: International Classification of Diseases Version 9
ICD-10: International Classification of Diseases Version 10
MK: Mann-Kendall test
MKKH: Modified Mann-Kendall test using Hamed and Rao variance correction approach
MOH: Ministry of Health Singapore
SGH: Singapore General Hospital
SNOMED CT: Systematized Nomenclature of Medicine Clinical Terms
SOC: Specialist Outpatient Clinic
