# Supplementary material for: Trends of chronic illness in emergency department admissions among elderly adults in a tertiary hospital over ten years
Source: BMC Health Serv Res. 2021 Dec 4;21:1305. doi: 10.1186/s12913-021-07309-z (PMC8645127; doi:10.1186/s12913-021-07309-z)
Supplement: Supplementary file 1 — Additional file 1: Table S1. List of chronic ACSC and the ICD-9 and ICD-10 Codes. Description of data: List of ICD-9 ad ICD-10 codes used to identify chronic ACSC. Figure S1. Geographical distribution of SGH emergency admission patients according to planning areas in Singapore. Description of data: Numbers show the total number of patients from the specific planning area from 2008 to 2017. [file 12913_2021_7309_MOESM1_ESM.pdf]

Article title: Trends of Chronic Illness in Emergency Department Admissions among Elderly Adults in a Tertiary Hospital over Ten Years

Journal name: Canadian Journal of Emergency Medicine

Authors: Zhongxun Hu[1], Fahad Javaid Siddiqui, MBBS, MSc[2], Qiao Fan, PhD[3], Sherman WQ Lian[4], Nan Liu, PhD[5], Marcus EH Ong, MBBS, MPH[6]

1. Duke-NUS Medical School, Singapore; email: zhu@u.duke.nus.edu (Corresponding author)
2. Prehospital and Emergency Research Centre, Health Services & Systems Research, Duke-NUS Medical School, Singapore
3. Center for Quantitative Medicine, Duke-NUS Medical School, Singapore
4. Department of Emergency Medicine, Singapore General Hospital, Singapore
5. Health Services & Systems Research, Duke-NUS Medical School, Singapore
6. Department of Emergency Medicine, Singapore General Hospital, Singapore; Health Services and Systems Research, Prehospital and Emergency Research Center, Duke-NUS Medical School, Singapore

Table.S1 List of chronic ACSC and the ICD-9 and ICD-10 Codes

**Chronic ACSC**	**ICD-9 Codes**	**ICD-10 Codes**
Angina	411.1, 411.8, 413	I20, I240, I248, I249
Asthma	493	J45
Chronic obstructive pulmonary diseases (COPD)	491, 492, 494, 496, 466.0	J20, J40, J41, J42, J43, J44, J47
Congestive heart failure	428, 402.01, 402.11, 402.91, 518.4	I50, I110, J810
Diabetes complications	250.1, 250.2, 250.3, 250.8, 250.9, 250.0	E101, E131, E110, E130, E10641, E11641, E106, E116, E108, E118, E109, E119
Epilepsy	345	G40
Hypertension	401.0, 401.9, 402.00, 402.10, 402.90	I10, I119, I16
Pulmonary tuberculosis	011	A150, A155, A159
Non-pulmonary tuberculosis	012-018	A154, A156, A158, A17, A18, A19

Fig.S1 Geographical distribution of SGH emergency admission patients according to planning areas in Singapore[1]

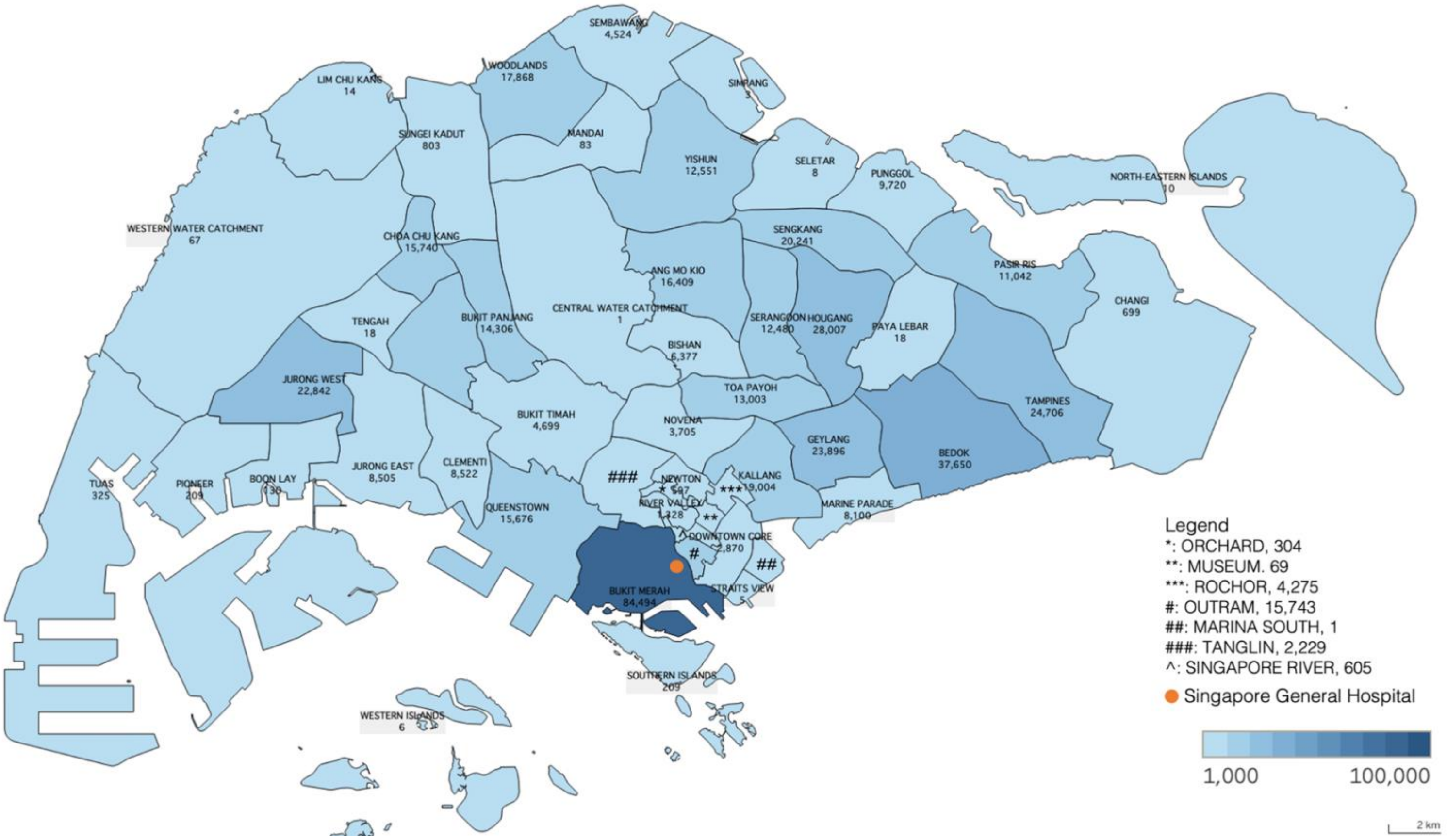

---

[1] Numbers show the total number of patients from the specific planning area from 2008 to 2017. The figure was generated using Tableau Desktop 2020.4. The figure contains information from Planning Area Census 2010 access on 4 May 2020 from Data.gov.sg which is made available under the terms of the Singapore Open Data Licence version 1.0. https://data.gov.sg/open-data-licence
